# Physical Quality Indicators and Mechanical Behavior of Agricultural Soils of Argentina

**DOI:** 10.1371/journal.pone.0153827

**Published:** 2016-04-21

**Authors:** Silvia Imhoff, Alvaro Pires da Silva, Pablo J. Ghiberto, Cássio A. Tormena, Miguel A. Pilatti, Paulo L. Libardi

**Affiliations:** 1 Consejo Nacional de Investigaciones Científicas y Técnicas, Esperanza, Santa Fe, Argentina; 2 Departamento de Ciencias del Ambiente, Facultad de Ciencias Agrarias, Universidad Nacional del Litoral, Esperanza, Santa Fe, Argentina; 3 Departamento de Ciência do Solo, Escola Superior de Agricultura "Luiz de Queiroz", Universidade de São Paulo, Piracicaba, São Paulo, Brasil; 4 Departamento de Agronomia, Universidade Estadual de Maringá, Maringá, Brasil; Banaras Hindu University, INDIA

## Abstract

Mollisols of Santa Fe have different tilth and load support capacity. Despite the importance of these attributes to achieve a sustainable crop production, few information is available. The objectives of this study are i) to assess soil physical indicators related to plant growth and to soil mechanical behavior; and ii) to establish relationships to estimate the impact of soil loading on the soil quality to plant growth. The study was carried out on Argiudolls and Hapludolls of Santa Fe. Soil samples were collected to determine texture, organic matter content, bulk density, water retention curve, soil resistance to penetration, least limiting water range, critical bulk density for plant growth, compression index, pre-consolidation pressure and soil compressibility. Water retention curve and soil resistance to penetration were linearly and significantly related to clay and organic matter (R^2^ = 0.91 and R^2^ = 0.84). The pedotransfer functions of water retention curve and soil resistance to penetration allowed the estimation of the least limiting water range and critical bulk density for plant growth. A significant nonlinear relationship was found between critical bulk density for plant growth and clay content (R^2^ = 0.98). Compression index was significantly related to bulk density, water content, organic matter and clay plus silt content (R^2^ = 0.77). Pre-consolidation pressure was significantly related to organic matter, clay and water content (R^2^ = 0.77). Soil compressibility was significantly related to initial soil bulk density, clay and water content. A nonlinear and significantly pedotransfer function (R^2^ = 0.88) was developed to predict the maximum acceptable pressure to be applied during tillage operations by introducing critical bulk density for plant growth in the compression model. The developed pedotransfer function provides a useful tool to link the mechanical behavior and tilth of the soils studied.

## Introduction

Soil is the basis resource of natural and agricultural ecosystems. Only 22% (3.26 billion ha) of the total area of the planet is suitable for agriculture, and only 3% (450 million ha) has inherent high production capacity. In addition, the regeneration of certain components of soil, such as organic matter, requires prolonged periods exceeding several times the human life expectancy. These characteristics make soil a finite and fragile resource, and maintaining its functionality is a topic of general interest. Various indicators have been proposed to evaluate the physical quality of soils for crop production. These indicators should include soil properties that affect the growth of plant roots, such as temperature, water and oxygen availability, and properties that indicate the presence/absence of mechanical constraints imposed by the soil matrix. The indicator named “least limiting water range” (LLWR) [1], which integrates three of those soil properties, namely water and air availability and mechanical resistance to root penetration, has proved to be correlated with root growth and productivity of various crops and forest species [2–5]. The LLWR is strongly affected by soil characteristics such as texture, organic matter content (OM) and soil bulk density (Bd) [6,7]. The LLWR decreases with increasing Bd, which implies that it is directly affected by soil compaction [8,9].

The process of soil compaction has been widely described by the compression curve and two indicators are obtained from this curve, i.e. the pre-consolidation pressure (σ) and the compression index (CI). The former reflects the bearing capacity of the soil while the latter the resistance to deformation [10].

The type and magnitude of soil deformation depends on external factors that determine the applied stress as well as on soil physical and mechanical properties, of which texture, organic matter content, and water content exert the greatest influence [11]. Texture determines how easily soil particles are rearranged when certain stress is applied [12]. The content and type of organic matter determines the binding forces between particles and aggregates [13]. The content of water determines the magnitude of soil deformation when certain stress is applied because water controls soil particles movement. Therefore, texture, organic matter content, and water content controls the physical degradation that soils will undergo [14,15].

Previous studies verified that the physical degradation of soil alters LLWR, σ, and CI, and that these indicators may positively or negatively be related to the intrinsic properties of the soil. For instance, an increase in Bd causes an increase in σ and a decrease in CI and the LLWR [16,17]. Some researchers have demonstrated that critical values of the mentioned indicators are dependent on soil characteristics [6,12,15,16].

The Pampas Region of Argentina, which is the main cropping area of the country, occupies about 50 million hectares [18], with about 34 million hectares of agriculturally useful lands. These lands have mainly gently rolling or flat relief. The region of flat relief is called the Flat Pampa. Fertile soils (Mollisol), extended croplands and cultivated pastures characterize these lands. However, at present, most of the soils of the Pampas have some degree of degradation. Changes in soil hydraulic parameters, such as pores size, number and connectivity, hydraulic conductivity, water flow, and water infiltration, as well changes in soil structure characteristics, such as form, size, dry-aggregation degree, aggregates stability, soil resistance to penetration, and bulk density were measured for several authors to evaluate soils degradation [19–21]. In addition, some researches were developed to evaluate the LLWR response to several tillage systems [6,8,22].

On the other hand, few researches were developed to evaluate maximum bulk density and compactability, expressed as the mean slope of the ascending part of the proctor curve, of the semi-arid and rolling Pampas soils [23–25].

Few indicators with the capability of evaluating both soil properties that affect plant grow and the mechanical behavior of soils were developed worldwide [26]. The determination of relationships between the LLWR, σ, and CI and their dependence on intrinsic soil properties would be very useful to assess soil tilth. Information on these relationships, known as pedotransfer functions (PTFs) [27], is very scarce especially for soils of the Argentinian Flat Pampas. Thus, the objectives of this study are i) to assess soil physical indicators related to plant growth and to soil mechanical behavior; and ii) to establish relationships to estimate the impact of soil loading on the soil quality to plant growth for the most productive soils of the flat Pampas of Argentina.

## Materials and Methods

The study was carried out in private farms spread over the most productive soils of the province of Santa Fe (Argentina), which cover about 10 million hectares, localized between the parallels 28°01´S and 34°40´S and the meridians 62°82´W and 58°05´W The owners of the farms gave us permission to conduct soil sampling and studies in all sites, which in turn did not involve endangered or protected species. The climate of the region is sub-humid-humid meso-thermal (C2B'3rd') [28], with an average annual temperature of 16°C in the extreme south and 21°C in the northern. Annual rainfall ranges from 800 to 1000 mm.

The studied soils were developed from the pampean loess and are classified as Mollisols. Information obtained from soil maps (1:50000 scale), aerial photos, and satellite images provided by the National Agricultural Institute of Rafaela was used for choosing soils. The following soil series were selected: Typic Argiudoll Murphy series, Typic Hapludoll Santa Isabel series, Typic Argiudoll Chovet series, Entic Hapludoll Saforcada series, Typic Argiudoll Rafaela series, Typic Argiudoll Castellanos series, Typic Argiudoll Esperanza series, Aquic Argiudoll Humboldt series, Typic Argiudoll Rincón de Ávila series, Typic Argiudoll Recreo series, Typic Argiudoll Ramayon series, Typic Argiudoll Las Gamas series, Typic Argiudoll Margarita series, and Typic Argiudoll Villa Minetti series [29].

A wide range of texture, organic matter and soil bulk density is required to develop pedotransfer functions (PTFs). Thus, to achieve this variability, soil samples were taken in different horizons and management systems, which included natural condition, continuous agricultural use (soybean/wheat/maize; soybean/wheat), continuous livestock use (alfalfa/ maize/ryegrass) and mixed systems (wheat/soybean/ryegrass/alfalfa). These systems predominated in the region studied. In each soil and for each management, disturbed and undisturbed samples were collected in the horizons A and B. Disturbed samples (each sample was composed of 20 subsamples) were used to determine particle density (Pd) with a gas pycnometer [30], granulometry with a Bouyoucos hydrometer [31], and soil organic carbon (SOC) by the Walkley-Black method [32]. Two types of undisturbed samples were collected in horizons A and B from each soil series: 5 cm diameter by 5 cm height cylinders (n = 600, 5 per management system) for determining LLWR, and 2.5 cm height by 7.5 cm diameter cylinders (n = 600, 5 per management system) for determining compression curves.

Cylinders measuring 5x5 cm were used to measure the soil water retention curves (WRC), soil resistance to penetration curves (SRC) and the LLWR [3]. Samples were gradually saturated with deionized water during 48 h, separated into groups, and then placed on tension tables and low and high pressure chambers until reaching equilibrium [33]. The matric potentials (ψ) applied were: -1, -2, -3, -4, -5, -6, -8, -10, -20, -30, -60, -100, -300, -600, and -1500 kPa. In each sample, soil resistance to penetration was determined with an electronic penetrometer that had a cone with a 4 mm diameter base. Readings were taken in the center of the sample at constant speed (1 cm min^-1^).

PTFs adjustment was made according to the procedure proposed by Silva and Kay [1]. WRC data were adjusted using an exponential model [34]:

$$\theta =a\times {\left|\psi \right|}^{b}$$ (1)

or alternatively

Lnθ=Lna+b×Ln|ψ| (2)

where θ is volumetric water content (cm^3^ cm^-3^), |ψ| is matrix potential (kPa), and *a* and *b* are adjustment parameters. Eq 2 can be expressed as follows:

$$Ln\theta =a_{0}+a_{1}p_{1}+a_{2}p_{2}+\ldots a_{i}p_{i}+\left(b_{0}+b_{1}p_{1}+b_{2}p_{2}+\ldots b_{i}p_{i}\right)\;\times \;Ln\left|\psi \right|$$ (3)

where *a*_*i*_ and *b*_*i*_ are adjustment parameters and *p*_*i*_ are soil properties. Regression multiple procedures (SAS Institute, 1991) were performed to evaluate the effects of texture, organic matter (OM = 1.724_*_SOC) and soil bulk density (Bd) on *a*_*i*_ and *b*_*i*_ parameters. Nonlinear effects were evaluated by the procedure in which each variable is transformed into a family of power transformations and the residues from each regression were evaluated according to Mosteller and Tukey [35]. The power term that yields the lowest residue was selected.

Soil resistance to penetration (SR) data were regressed against θ and Bd using the following model [36]:

$$SR=c\;\times \;\theta^{d}\;\times {\;Bd}^{e}$$ (4)

or alternatively

LnSR=Lnc+d×Lnθ+e×LnBd (5)

where SR is the soil resistance to penetration (MPa), θ is volumetric water content (cm^3^ cm^-3^), Bd is bulk density (g cm^-3^), and *c*, *d*, and *e* are fitting parameters. The influence of granulometry, OM, and Bd was evaluated using a procedure similar to WRC, and the function can be expressed as follows:

$$\begin{array}{l} LnSR=c_{0}+c_{1}p_{1}+c_{2}p_{2}+\ldots \;c_{i}p_{i}+\left(d_{0}+d_{1}p_{1}+d_{2}p_{2}+\ldots \;d_{i}p_{i}\right)\;Ln\theta +\\ \;+\left(e_{0}+e_{1}p_{1}+e_{2}p_{2}+\ldots \;e_{i}p_{i}\right)\;LnBd \end{array}$$ (6)

where *c*_*i*_, *d*_*i*_, and *e*_*i*_ are adjustment parameters and *p*_*i*_ are soil properties. Nonlinear effects were evaluated with the same procedure as explained above.

Samples measuring 2.5x7 cm were also gradually saturated with distilled water, separated into groups, and then placed in tension tables and low pressure chambers of Richards until the drainage had stopped. Each group was subject to a matric potential (ψ: -10, -20, -30, -60, and -100 kPa) to establish a moisture gradient. After equilibration, samples were subject to a uniaxial compression test using an automated consolidation system that transfers deformation readings experienced by the soil directly to a computer. Pressure values (25, 50, 100, 200, 400, 600, 800, 1000, 1300, and 1600 kPa) were applied consecutively for five minutes, which was sufficient time to reach 90% of maximum deformation according to Imhoff et al. [16]. After pressure release, samples were dried in an oven at 105°C for 24 hours to determine the dry mass value (g) of each sample. Soil bulk density values (Bd) were calculated based on the dry mass value (g) and corresponding volumes at each pressure level [37]. The soil bulk density value prior to application of selected pressures was called the initial soil bulk density of soil (Bd_i_). To calculate the compression index (CI) and pre-consolidation pressure (σ), a program developed using Mathcad software was used [16].

Compression curve was described by using the model proposed by McNabb and Boersma [38]. This model predict Bd as a function of the applied stress (σ_a_), initial Bd and water content, which is expressed in terms of degree of saturation. The equation is:

$$LnBd=Ln\left(Bd_{0}\times Bd_{n}\right)-\left[a+b\times \sigma_{a}+c\times \left(Bd_{c}\times Bd_{0}\right)+e\times {\left(\theta \right)}^{g}+f\times {\left(\theta \right)}^{g}\times \left(1-\text{exp}\left(-d\times \sigma_{a}\times \left(1-\theta^{g}\right)\right)\right)\right]$$ (7)

Where: Bd_0_ is Bd at zero applied stress; Bd_n_ is the quotient between initial Bd (Bd_i_) and average Bd, and normalizes Bd_0_ for differences in Bd_i_; Bd_c_ is equal to ((Bd_n_-1)_*_Bd_0_) and adjusts the compression curve for differences in Bd_i_ of each sample; σ_a_ is de applied stress (kPa); θ is defined as (1-θ_SD_), and takes into account for the effect of water content; θ_SD_ is the degree of saturation; *a*, *b*, *c*, *d*, *e*, *f*, and *g* are the estimated parameters. Coefficient *g* was added to take into account for a possible nonlinear effect of degree of saturation.

The influence of water content, organic carbon, texture, and soil bulk density on the water retention curve, penetration resistance curve, least limiting water range, compression index, pre-consolidation pressure, and compression curve was quantified by multivariate and nonlinear regression techniques with SAS software [39]. The presence of multicollinearity problems was assessed with VIF (Variance Inflation Factor) [40]. In the developed models, only variables with VIF<10 were included.

## Results and Discussion

The description of the properties of the soils included in the determination of WRC, SR, and LLWR is shown in Table 1.

**Table 1:** Soil properties of samples used to describe the soil water retention and soil resistance to penetration curves and the least limiting water range (n = 600).

Variable	Mean±SD	Coefficient of Variation (%)	Minimum	Maximum
**OM (%)**	2.65±0.96	17	0.8	5.0
**Clay (%)**	27±10	39	12	54
**Sand (%)**	18±18	36	3	73
**Silt (%)**	55±15	27	16	76
**Bd (g cm**^**-3**^**)**	1.37±0.09	6.8	1.01	1.69
**SR (MPa)**	1.97±1.43	73	0.21	7.07
**WC (g g**^**-1**^**)**	0.26±0.10	39	0.04	0.49

OM: organic matter; Bd: soil bulk density; SR: soil resistance to penetration; WC: water content.

In agreement with the majority of published PTFs, soil texture, bulk density and OM were used as predictors due to these properties are available from soil surveys or may be easily measured in the laboratory [6,15,27]. Wide ranges of the mentioned soil properties are needed to achieve high generality for the predictions [6]. The studied soils had wide variability in their soil physical properties. Thus, the principle of getting wide variability of soil properties for obtaining FTPs was achieved.

### Soil physical indicators related to plant growth

The LLWR concept defines a range of soil water contents within which root and shoot growth is least limited from the physical conditions of the soil [2–5]. The first step for calculating the LLWR consists on determining the WRC and the SR curve because they allow calculating the upper and lower limits of the LLWR. The former is determined by the lower value of water content between the water content at field capacity and water content corresponding to 10% air-filled porosity. The latter is determined by the higher value of water content between the water content at wilting point and water content corresponding to 2 MPa of SR. All these values must be known for the range of bulk densities that occur under field conditions for different type of soils [1].

The parameters of the model of WRC are shown in Table 2.

**Table 2:** Parameters of the model of the retention water curve. *Lnθ* = *a*_0_ + *a*_1_*Lnclay* + *a*_2_*LnOM* + (*b*_0_ + *b*_1_*Lnclay*) × *Ln*|*ψ*|.

Parameter	Estimated Value±SE	*t* value	Pr > |t|
**a0**	-0.749±0.103	-7.29	<0.0001
**a1**	0.021±0.030	0.69	0.0049
**a2**	0.042±0.012	3.57	<0.0004
**b0**	-0.352±0.025	-14.27	<0.0001
**b1**	0.066±0.008	8.82	<0.0001

θ = volumetric water content (cm^3^ cm^-3^); OM = organic matter (%); clay (%); ψ = water potential (kPa). *F*-value of the model = 749.8; significant at *P* < 0.0001 probability level; *R*^*2*^ = 0.91.

The WRC model explained 91% of the variability of the soil water retention data (S1 Fig). Clay and OM content had a nonlinear effect on soil water content that was described by a logarithmic (Ln) transformation of both variables. An interaction term between Lnclay and Ln|ψ| was verified. Nonlinear effects of clay and organic matter on Lnθ were also found by other authors [1,3–6]. These results were expected from the wide range of clay and organic matter content of the soils. In addition, the influence of clay and organic matter on water retention had been quantified by several authors [6,41–44]. Moreover, most of the pedotransfer functions (PTFs) include OM and texture as predictor variables.

**Fig 1:**
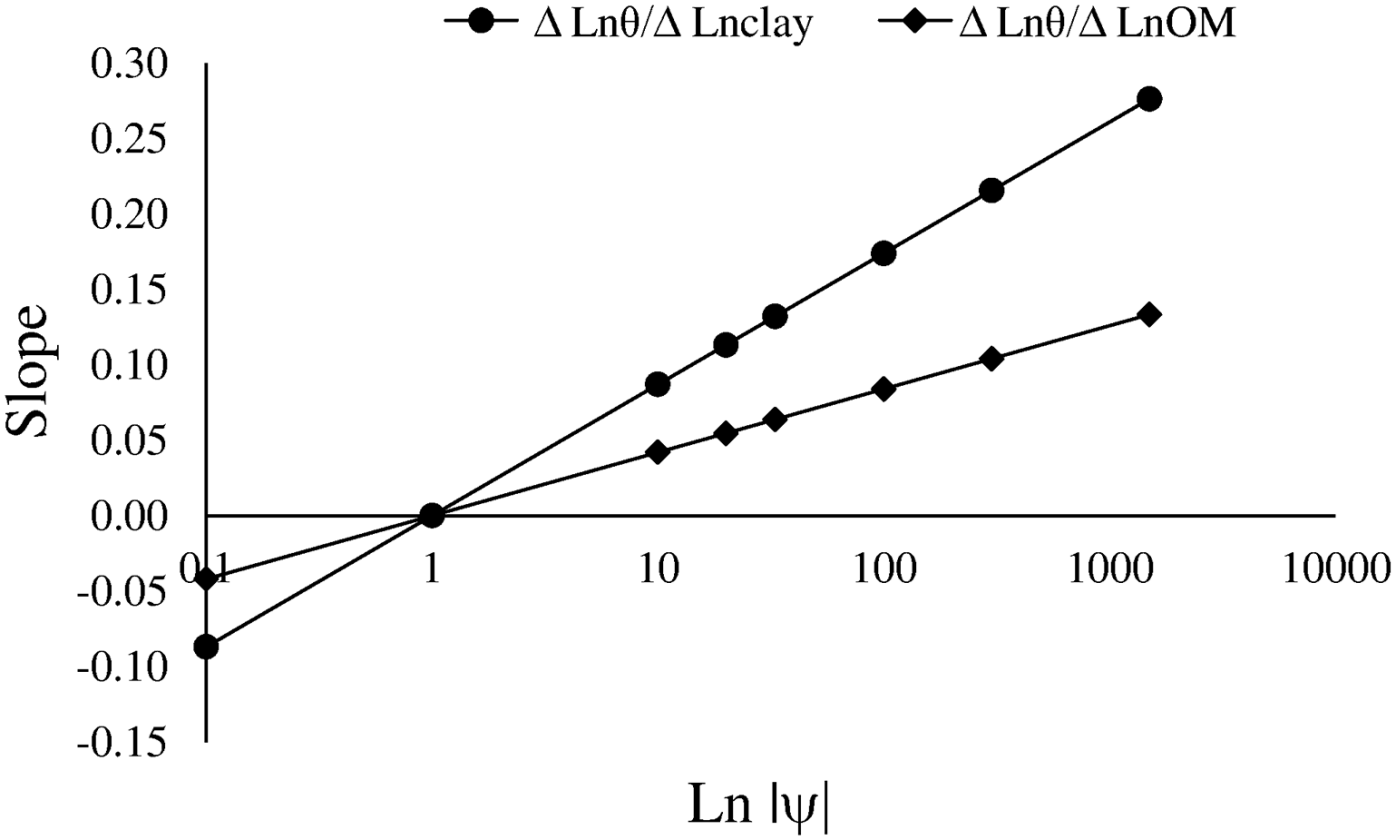
Slope of the relationships Ln volumetric water content (Lnθ) x Ln clay content (Lnclay) and Ln volumetric water content (Lnθ) x Ln organic matter (LnOM) as function of Ln water potential (Ln|ψ|).

On the other hand, Bd did not significantly affect model fit. However, Bd is related to clay and OM, which may explain the result. The negative correlation between clay and Bd, and between OM and Bd has been demonstrated repeatedly. Silva and Kay [6] used the simple slope of the relationships Lnθ x Lnclay, and Lnθ x LnOM to describe the features of the interactions. The simple slope of the relationships is shown in Eqs 8 and 9 and Fig 1.

ΔLnθ/ΔLnclay=0.021+0.066×Ln|ψ| (8)

ΔLnθ/ΔLnOM=0.042×Ln|ψ| (9)

The effect of clay and OM on soil water retention increases with decreasing ψ (i.e. increasing |ψ|), even though the effect of clay is greater than that of OM. Similar results were found by Silva and Kay [6]. Close to saturation (|ψ| = 0.01 kPa) both variables have negative effect on water retention, which was reversed at |ψ| = 1 kPa. This result suggest that clay and OM increase water retention by affecting soil pores of radius < 150 μm. Silty clay loam soils of the Pampas have very low volume of large macropores (> 150 μm), which in turn are very unstable [21]. This characteristic is probably responsible for the negative influence of clay and OM on water retention at high water potential (close to saturation). Furthermore, Rawls et al. [42] highlight the existence of contradictory reports about the effects of clay and OM content on soil water retention. The authors concluded that differences were due to the effect of OM content in soil water retention depends on the textural composition of the soil.

The parameters of the model of SR are shown in Table 3.

**Table 3:** Parameters of the model of soil resistance to penetration. *LnSR* = *c*_0_ + *c*_1_*clay* + *c*_2_*OM* + (*d*_0_ + *d*_1_*clay*) *Lnθ* + (*e*_0_) *LnBd*.

Parameter	Estimated Value±SE	*t* value	Pr > |t|
**c0**	-4.877±0.283	-17.21	<0.0001
**c1**	-0.017±0.010	-1.74	0.0082
**c2**	0.423±0.024	17.39	<0.0001
**d0**	-1.725±0.295	-5.94	<0.0001
**d1**	-0.050±0.012	-4.28	<0.0001
**e0**	5.604±0.334	16.79	<0.0001

Clay (%); OM = organic matter (%); θ = volumetric water content (cm^3^ cm^-3^); Bd = g cm^-3^. *F*-value of the model = 229.6; significant at *P* < 0.0001 probability level; *R*^*2*^ = 0.84.

The SR model explained 84% of the variability of SR data (S2 Fig). Soil water content (θ) had a negative effect on SR, while soil bulk density (Bd) had a positive effect. An interaction between clay and soil water content was determined, which implies that the effect of θ on SR depends on clay content. Organic matter content also had significant effect on the model in agreement with the finding of other authors [6,45].

**Fig 2:**
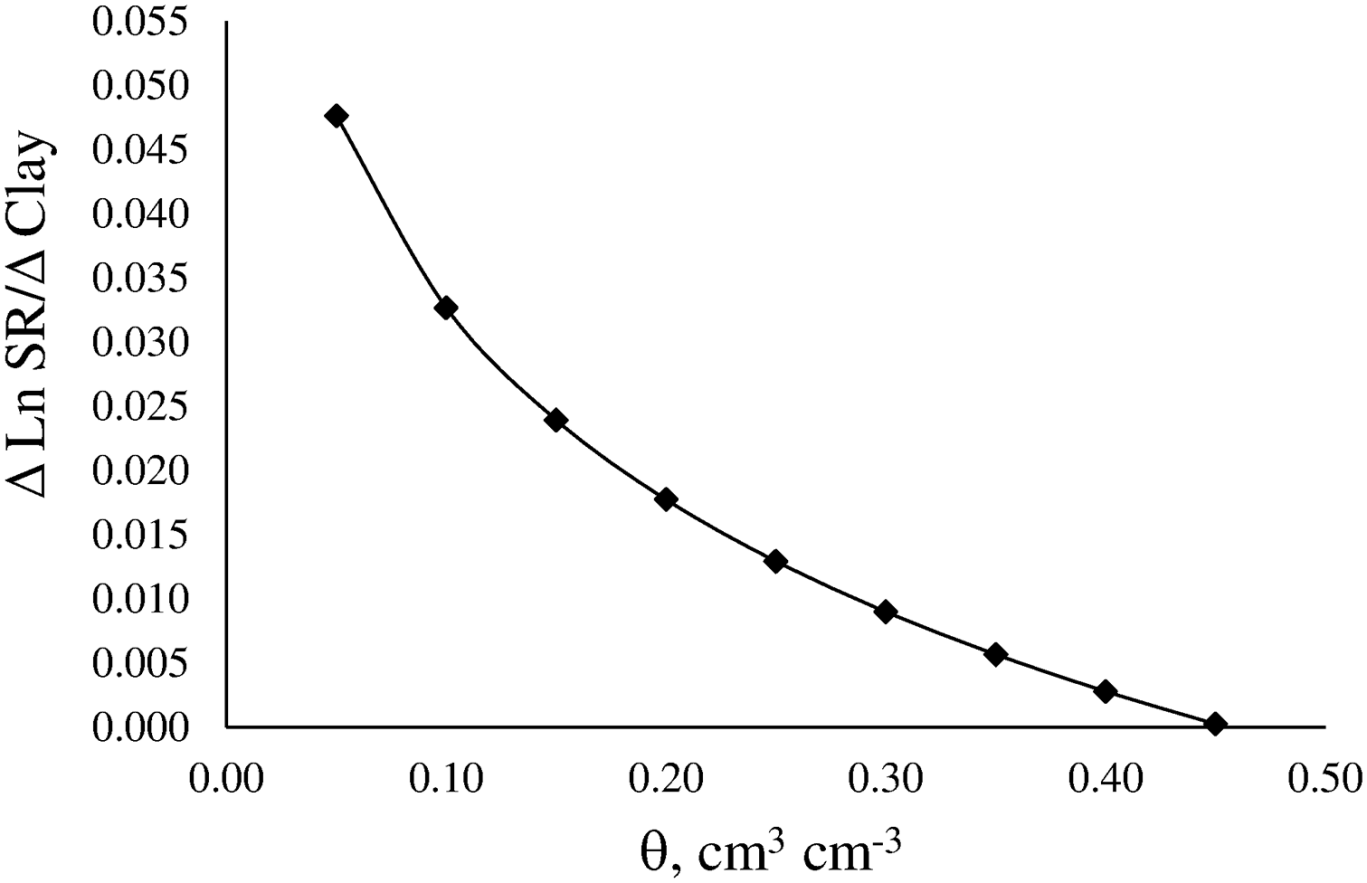
Slope of the relationships Ln soil resistance to penetration (Δ LnSR) x clay content (Δ clay) as function of volumetric water content (θ).

The simple slope of the relationship between LnSR and clay is shown in Eq 10 and Fig 2.

ΔLnSR/ΔClay=−0.017−0.050×Ln|θ| (10)

The Δ LnSR/Δ Clay decreases with increasing water content and becomes negative at high values of soil water content. Similar result was found by Silva and Kay [6].

**Fig 3:**
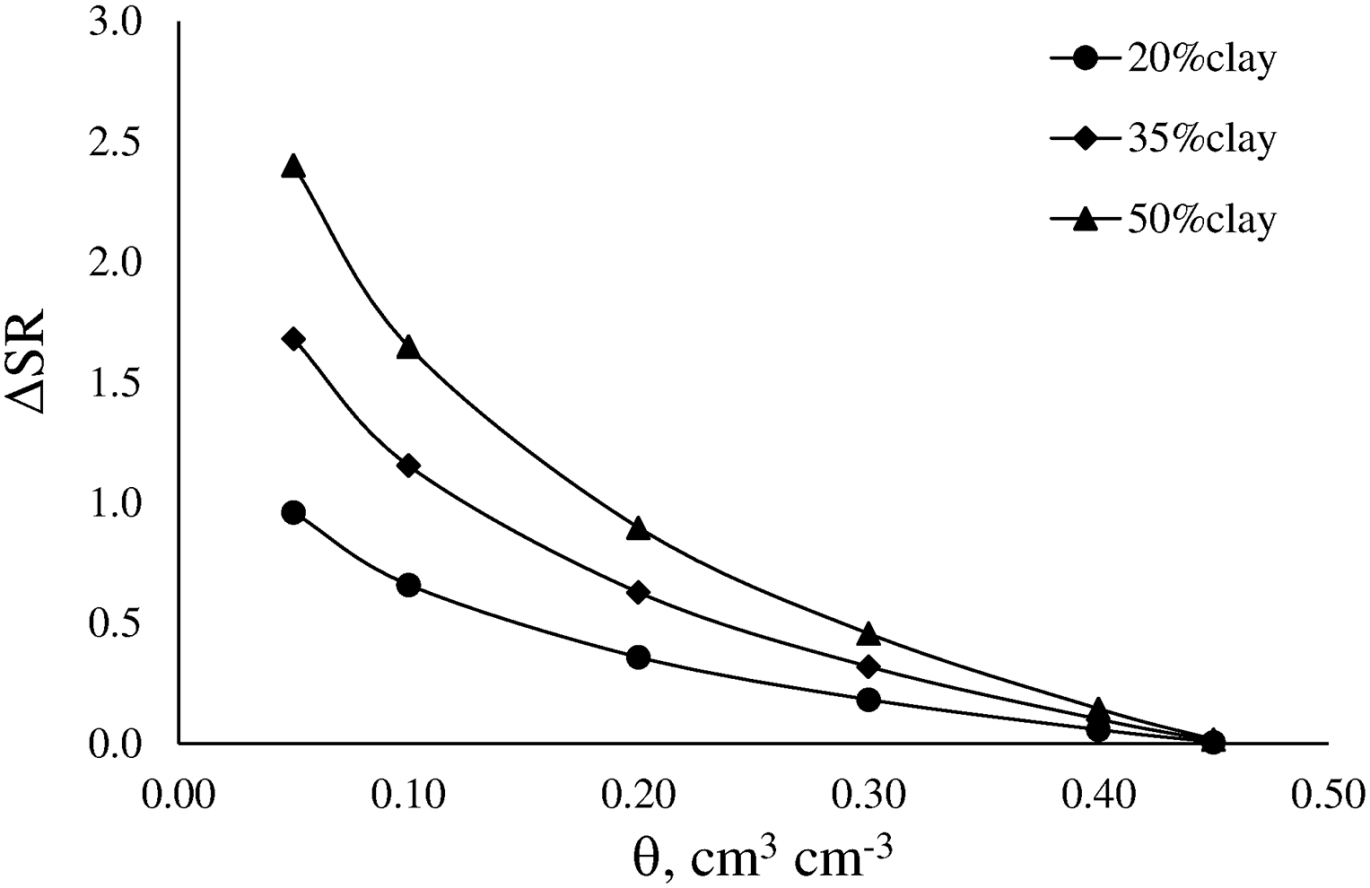
Effect of clay content on the variation of soil resistance to penetration (Δ SR) as function of volumetric water content (θ).

SR increases with increasing clay content and the magnitude of the effect decreases as water content increases (Fig 3).

This effect may be associated with the increase of the effective stress. SR is related to the interaction between relative saturation of the soil and water potential, i.e. the effective stress. The effective stress is determined by two components: at high soil water content effective stress is mainly produced by the prevailing water potential in water-filled pores; at low soil water content effective stress is mainly produced by isolated water films around soil particles [44]. Therefore, this component is notably influenced by soil texture, mainly by clay content. Ours results confirm the findings of Vepraskas [44]. To and Kay [43] have already mentioned that soil texture influences SR by controlling changes in soil water content and thus, in the effective stress. On the other hand, the increase of soil water content causes the degree of saturation to increase and water potential in water-filled pores to change. As a result, the effective stress decreases, which in turn causes SR to decrease.

**Fig 4:**
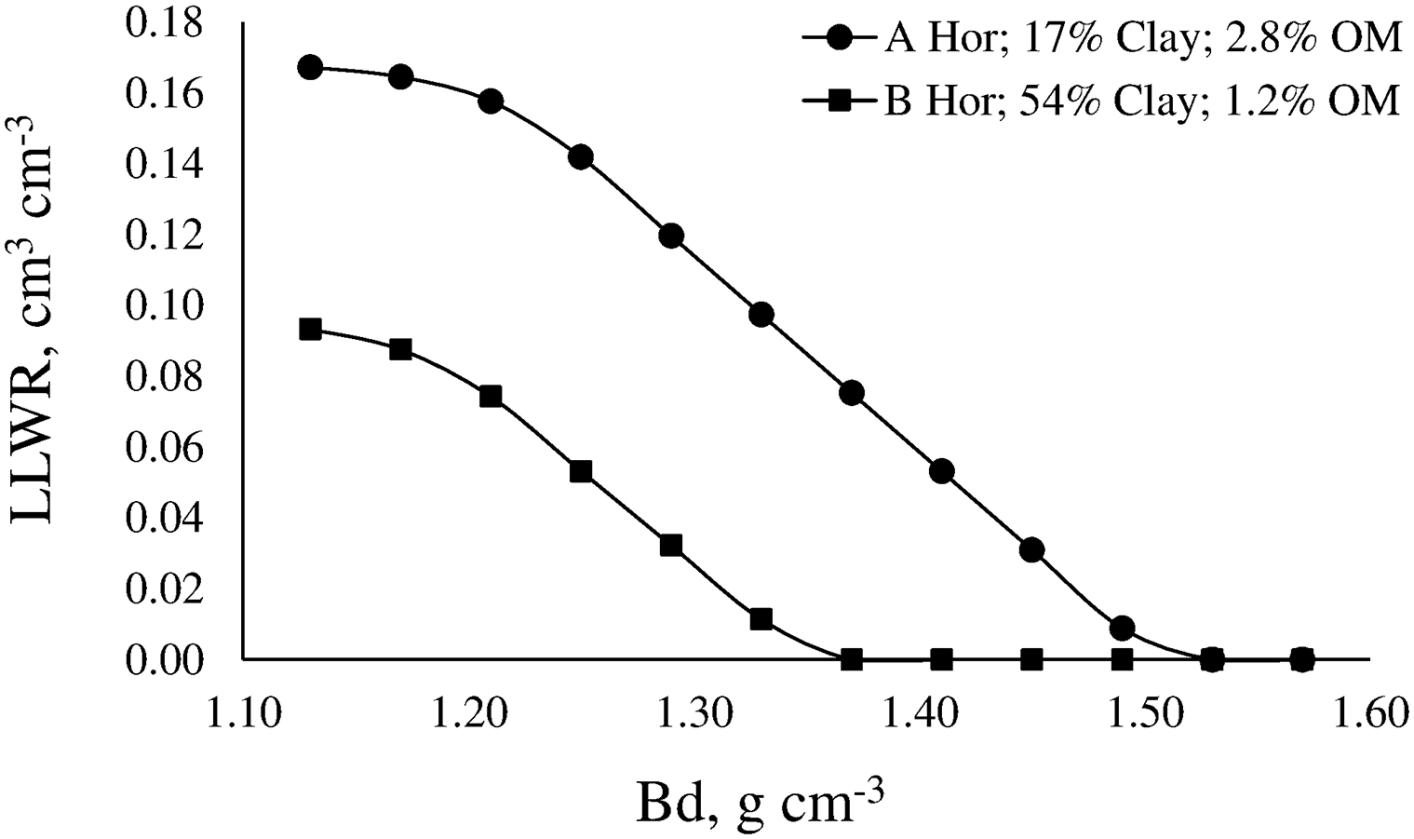
Least limiting water range (LLWR) for a soil with 17% clay and 2.8% organic matter (A horizon), and for a soil with 54% clay and 1.2% organic matter (B horizon).

The models of WRC and SR were used to determine LLWR. Fig 4 shows the LLWR for the A horizon of a Typic Argiudoll Santa Isabel series of loamy texture, and the B horizon of a Typic Argiudoll Esperanza series of silty-clayed texture.

Regardless of soil characteristics, the increase of Bd caused the LLWR to decrease due to soil water content associated with the critical value of SR = 2.5 MPa (θSR) increased, and soil water content associated with the critical value of air-filled porosity equal to 10% (θAFP) decreased in both soils. The loamy soil had water content at field capacity (θFC) slightly lower than the silty-clayed soil (0.33 vs 0.34 cm^3^ cm^-3^). Also, the former had water content at permanent wilting point (θPWP) lower than the latter (0.20 vs 0.23 cm^3^ cm^-3^). As consequence, the amount of available water is slightly greater in the loamy soil than in the silty-clayed soil. However, both soils have very different LLWR. In fact, the LLWR in the silty-clayed soil is much narrower than in the loamy soil due to θSR has acted as lower limit across all range of Bd. In addition, critical soil bulk density (Bd_c_) was reached at Bd = 1.38 g cm^-3^ in the silty-clayed soil while it was reached at Bd = 1.53 g cm^-3^ in the loamy soil, which demonstrates the better physical condition of this soil.

**Fig 5:**
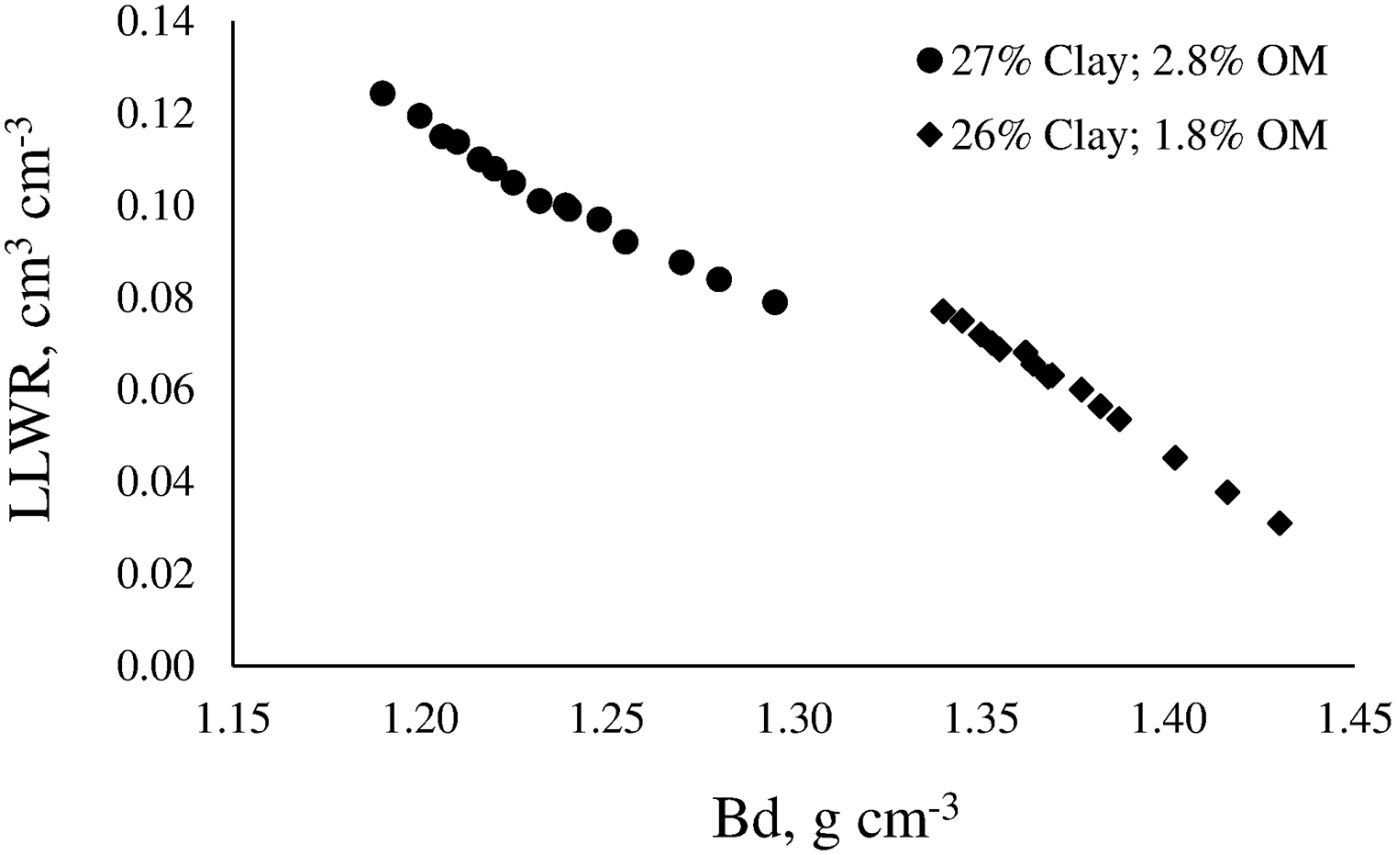
Variation of the least limiting water range (LLWR) for two soils with similar clay content and different organic matter (OM) content.

The magnitude of the LLWR was also conditioned by the content of OM (Fig 5) and clay.

**Fig 6:**
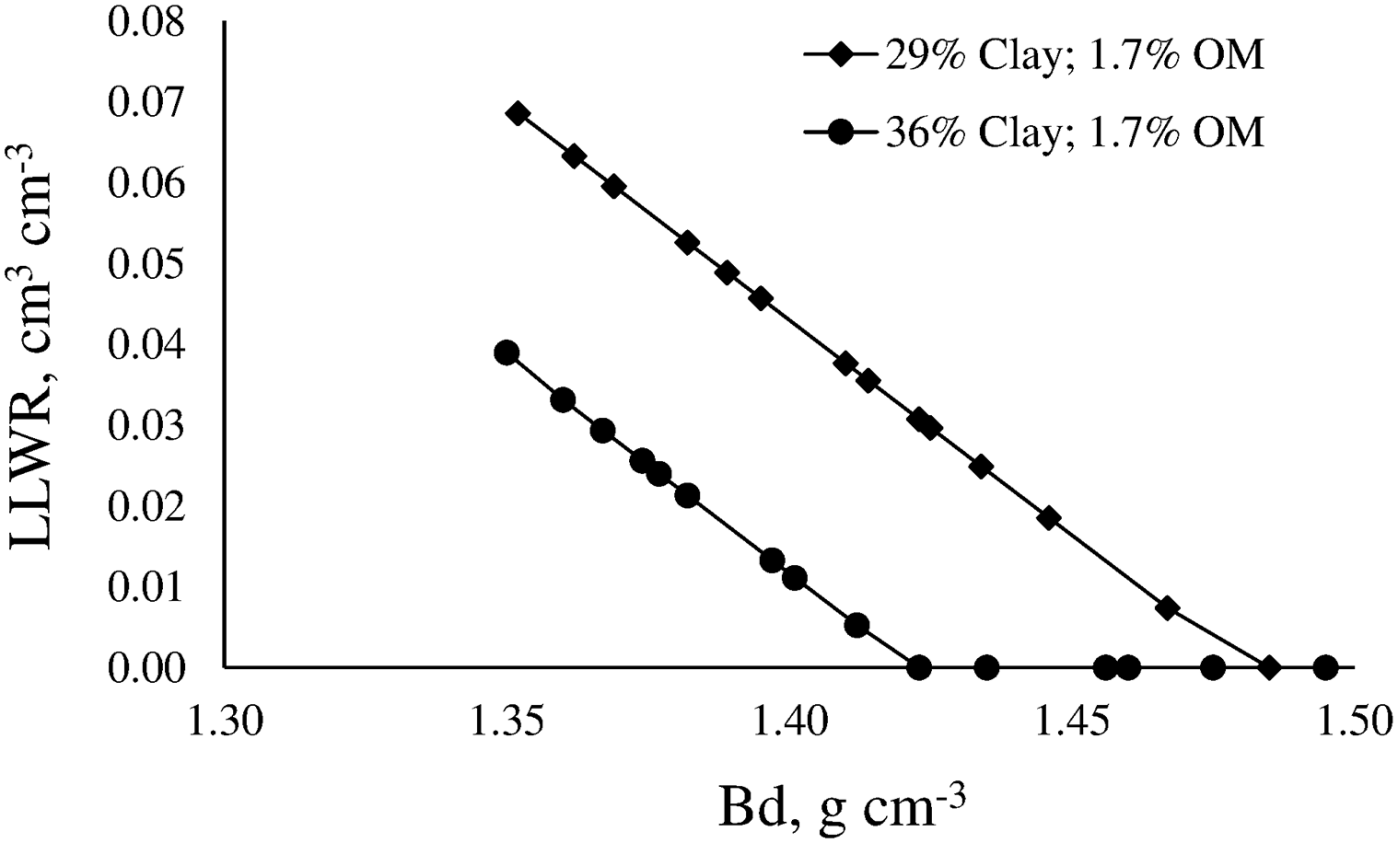
Variation of the least limiting water range (LLWR) for two soils with similar organic matter (OM) content and different clay content.

Soils with lower OM content seem to show a slightly higher rate of decline of the LLWR than soils with higher OM. Therefore, it is expected that the increase of soil compaction causes greater reduction of the LLWR in soils with lower OM. However, the major effect of OM in the LLWR is indirect by decreasing Bd. Similar effect of OM on Bd was demonstrated in several researches [6,43,45,46]. Moreover, To and Kay [43] have mentioned that SR increased with increasing organic carbon when texture and Bd were constant, which in turn is in agreement with ours results (Table 3). These authors stated that this behavior was consistent with increased cementation within microaggregates by soil OM. The LLWR variation with Bd of two soils with contrasting clay content and similar OM is shown in Fig 6.

The main effect of clay on LLWR consist on reducing the magnitude of the LLWR. The rate of decline is similar in both soils and reduction occurs across the same range of Bd. This behavior may be associated with the negative effect of clay on air-filled porosity, which causes the LLWR´s upper limit to decrease, and the positive effect of clay on soil resistance (Fig 3), which causes the LLWR´s lower limit to increase. These findings are in agreement with the results found in several researchers carried out in temperate soils [6,43].

**Fig 7:**
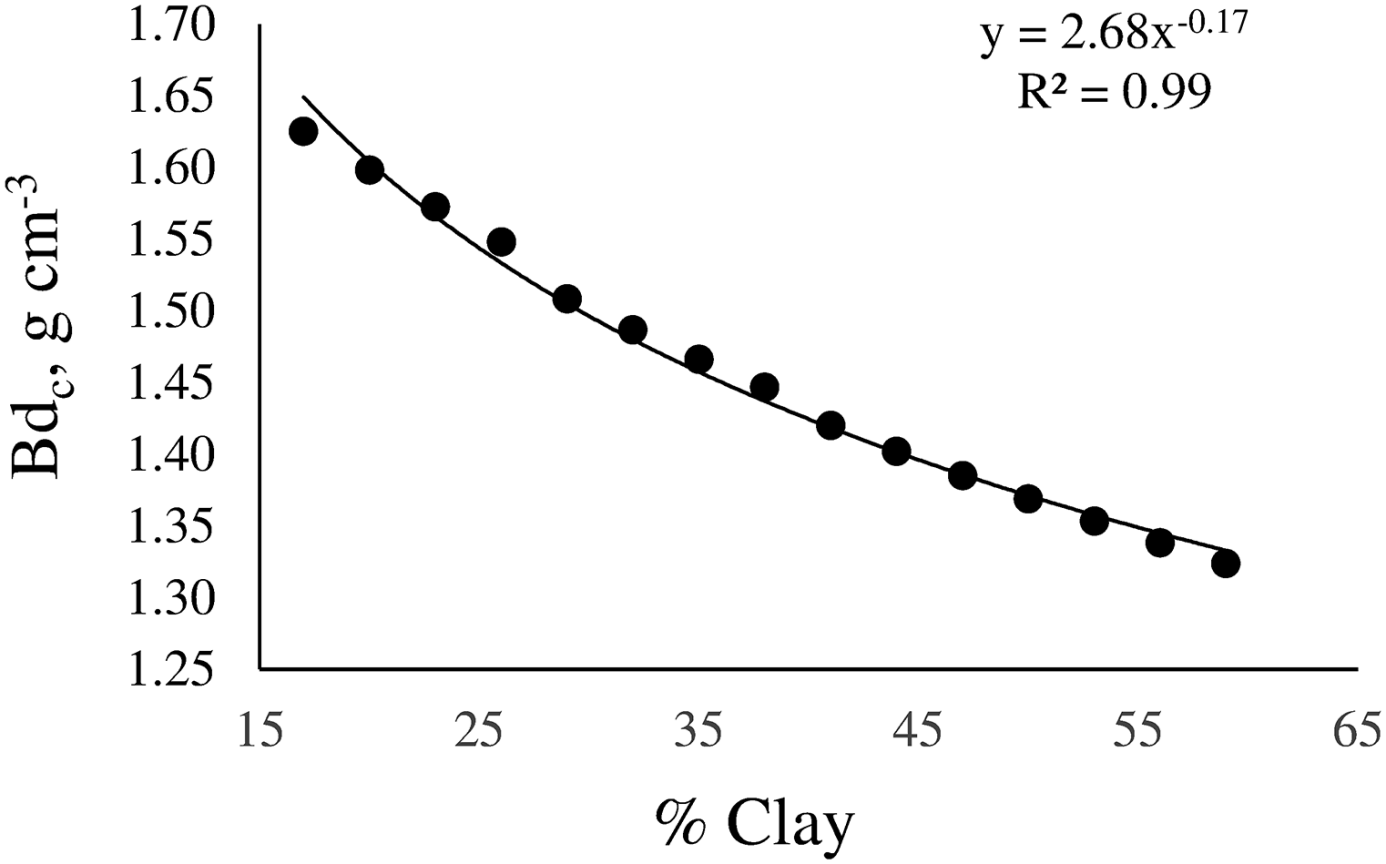
Variation of the critical soil bulk density (Bd_c_) as a function of clay content.

Results demonstrate that soil properties determine the magnitude of the LLWR. The main implication of this finding is that Bd_c_ strongly depends on soil texture, especially on clay content. Critical soil bulk density decreases with increasing clay content. The better fit was reached with an exponential function (Fig 7).

This behavior implies that an increase in soil compaction in clayed soils would involve inadequate physical conditions for plant growth in a Bd gradient lower than in sandy soils. Values of Bd_c_ calculated from the LLWR are in agreement with threshold values of Bd calculated by other authors [47,48].

The overall results indicate that the developed PTFs can be used to determine the LLWR of a specific soil as well as to describe the relationships between the LLWR and soil properties. Moreover, the developed PTFs allow the estimation of Bd_c_ for plant growth from the clay content of the soil.

### Physical indicators related to soil mechanical behavior

Statistical moments of the analyzed variables and parameters obtained from the compression curves are presented in Table 4. The large amplitude of the variation of the studied parameters is most likely determined by the variability of texture and OM.

**Table 4:** Statistical moments of the soil physical properties and parameters obtained from the compression curves. n = 600.

Variable	Mean±SD	Coefficient of Variation (%)	Maximum	Minimum
**OM (%)**	2.92±0.47	16	4.03	1.98
**WC**_**g**_ **(g g**^**-1**^**)**	0.26±0.06	22	0.35	0.17
**Clay (%)**	24±6.36	27	34	10
**Sand (%)**	19±17.17	93	68	3
**Silt (%)**	57±12.15	21	70	21
**Bd (g cm**^**-3**^**)**	1.35±0.11	8.19	1.56	1.06
**Pd (g cm**^**-3**^**)**	2.53±0.03	1.32	2.60	2.46
**σ kPa)**	157±68.28	43.51	371	32
**CI**	0.25±0.04	17.42	0.35	0.17

OM: organic matter, WC_g_: gravimetric water content, Bd: soil bulk density, Pd: soil particle density, σ preconsolidation pressure, CI: compression index.

Pre-consolidation pressure and compression index are commonly used as indicators of the vulnerability of soils to compaction damage. Average values and range of variation of σ and CI were lower or similar to those obtained by other authors [12,15].

Differences in CI may be associated with differences in soil texture and organic matter of the soils studied. Table 5 shows the properties that have conditioned CI and the parameters of the fitted model, which explains 77% of the variability of the data.

**Table 5:** Parameters of the compression index (CI) model. *CI* = *a* + *bLnWC*_*g*_ + *cBd* + *dLnClSi* + *eLnOM*.

Parameter	Estimated Value±SE	*t* value	Pr > |t|
**a**	0.431**±**0.06	7.27	<0.0001
**b**	0.119**±**0.01	8.74	<0.0001
**c**	-0.127**±**0.03	4.97	<0.0001
**d**	0.053**±**0.01	5.74	<0.0001
**e**	-0.076**±**0.02	-4.69	<0.0001

WC_g_: gravimetric water content (g g^-1^); Bd: bulk density (g cm^-3^); ClSi: clay+silt (%); OM: organic matter (%). *F*-value of the model = 58.66; significant at *P* < 0.0001 probability level; *R*^*2*^ = 0.77.

Soil CI was negatively related to soil Bd and OM. Soil strength is a results of the total contact points between particles and the shear resistance per contact point [11]. Friction forces between soil particles increases with increasing Bd, which explains the negative relationship determined in this research and others [13,15,49]. The effect of OM was associated with the increase in cohesive forces and the reduction of the water content range in which soil exhibits plastic properties [50]. The effect of OM seems to depend on the content of water that soil has at the time of compaction [51]. Also, OM increases the rigidity of the pore system and the bond strength at the contact points, which contribute to decrease CI. Similar results were found for other authors [52,53].

On the other hand, CI was positively related to WC_g_ as well as clay plus silt content. Soil particles can easily move when are surrounded by waters films. Therefore, soil becomes more prone to undergone deformation as soil water content increases. Soil deformation is also facilitated by the increase of clay content, which are in agreement with many other studies carried out in laboratory [16,45].

**Fig 8:**
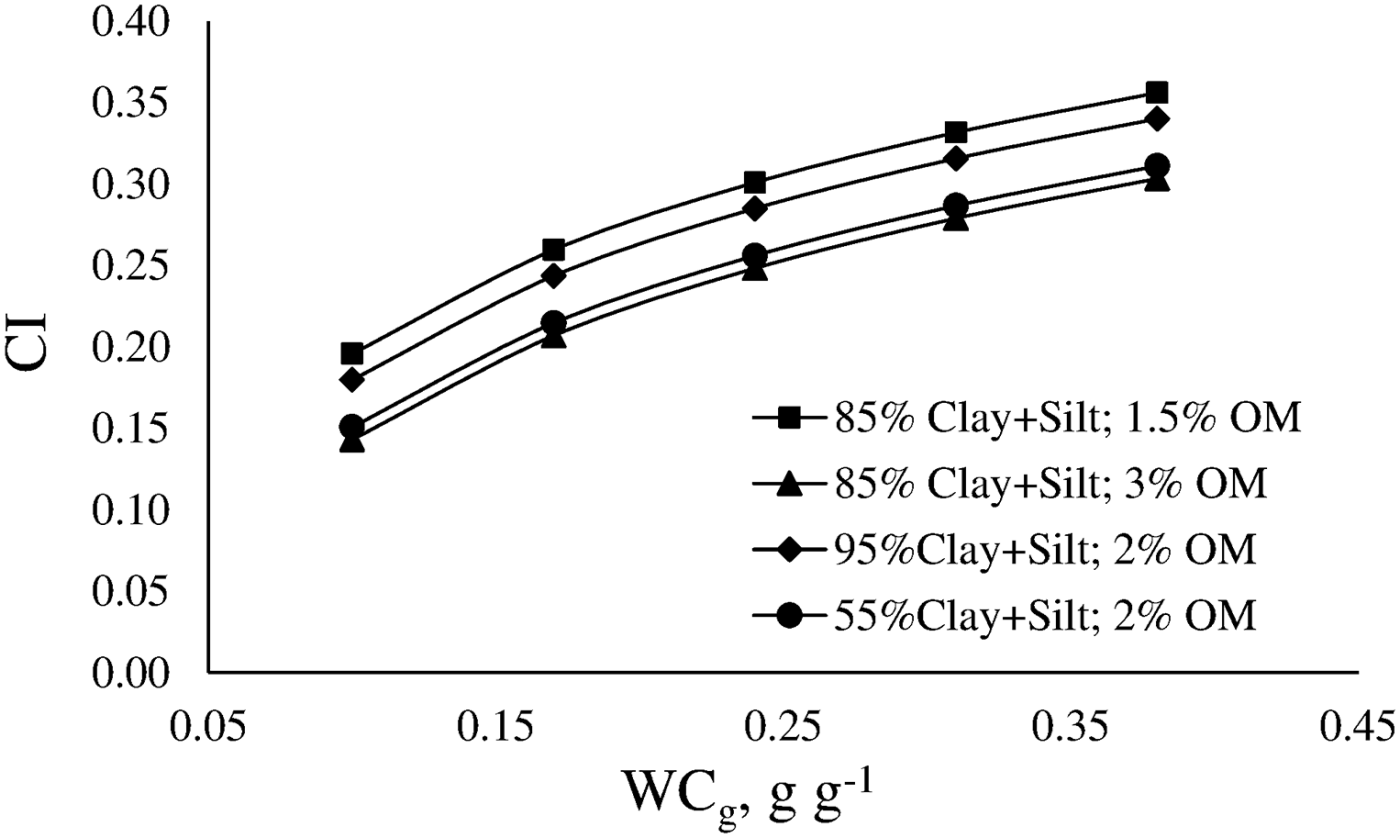
Variation of the compression index (CI) as a function of gravimetric water content (WC_g_) for several combination of Clay+Silt content and organic matter content (OM).

Soils of the Flat Pampa have predominance of clay and very fine silt. Clay fraction is mainly constituted by 2:1 non-expandable phyllosilicates (illite), whereas silt fraction is mainly constituted by phytoliths and other bioliths. The percentage of these materials in the silt fraction may reach 42% [54]. Phytoliths consist of a very porous gel of amorphous silica with variable amount of water and organic matter occluded in the criptopores. The particles of this material have shape of elongated rods, low density and very high fragility, which cause phytoliths to be easily broken. Illitic-type minerals have sheet-shaped and exhibit low shrink-swell capacity [55]. The stated characteristics of both fractions facilitates the movement and the tightened rearrangement of the particles when the applied stress exceeds the soil strength. Moreover, both the content and the special characteristics of clay and silt may be responsible for the values of CI. Días-Zorita and Grosso [24] have also found that the compactability of Mollisols from the Rolling Pampa increased with increasing clay and silt content. Other authors found that clay and silt contents have also conditioned CI [45]. Fig 8 shows the effects of WC_g_ on CI for different combinations of texture and OM, and for a value of Bd = 1.25 g cm^-3^.

Soil WC_g_, texture and OM affect nonlinearly the values of CI. CI shows a degressive increase with increasing WC_g_. For a certain value of WC_g_, CI increases with decreasing OM. Similarly, CI increases with increasing clay+silt content. Therefore, soil compaction would have stronger negative consequences in soils with fine-texture and lower OM content. Soils with low OM content showed low capacity of recovering the initial state after being subjected to compaction, i.e. low resilience [56].

The range of variation of σ (Table 4) suggests that evaluated soils were not subject to excessively high loads [11]. In general, agricultural machinery applies pressures ranging from 70 to 350 kPa, while transport equipment applies pressures of up to 800 kPa. Animals can apply pressures ranging from 50 to 350 kPa according to their weight [57]. In the Pampas region non-heavy agricultural machinery is used for plowing, no-till and harvesting crops, which explain the findings. In addition, the strong intensification of the livestock systems has begun few years ago. Therefore, pressures applied by the animals have not caused severe additional compaction to the soils.

The relationship between pre-consolidation pressure (σ) and soil properties is shown in Table 6.

**Table 6:** Parameters of the pre-consolidation pressure model (σ). *σ* = *a* + *bLnOM* + *cLnCl* + *dLnWC*_*g*_.

Parameter	Estimated Value±SE	*t* value	Pr > |t|
**a**	-402.849±56.21	-7.17	<0.0001
**b**	64.466±22.36	2.88	<0.005
**c**	45.297±11.61	3.90	<0.0002
**d**	-250.874±16.84	-14.89	<0.0001

OM: organic matter (%); Cl: clay (%); WC_g_: gravimetric water content (g g^-1^). *F*-value of the model = 75.77; significant at *P* < 0.0001 probability level; *R*^*2*^ = 0.77.

Pre-consolidation pressure (σ) decreases with increasing WC_g_ of the soil, and increase with increasing OM and clay content. These results in general agree with the findings of Imhoff et al. [16] and Saffih-Hdadi et al. [17] but disagree with the findings of Keller et al. [10] and Arthur et al. [13]. These authors attributed the lack of correlation between σ and texture to differences in soil packing state due to previous anthropogenic activities. Several researches reported σ increased with increasing Bd [16,17] whereas other found the opposite effect [13]. In this study σ was not related to Bd. However, it is well kwon that Bd is conditioned by soil texture and organic matter. Therefore, the influence of Bd could have been covered by the variability of texture and organic matter of the soils, and the diversity of management system studied.

**Fig 9:**
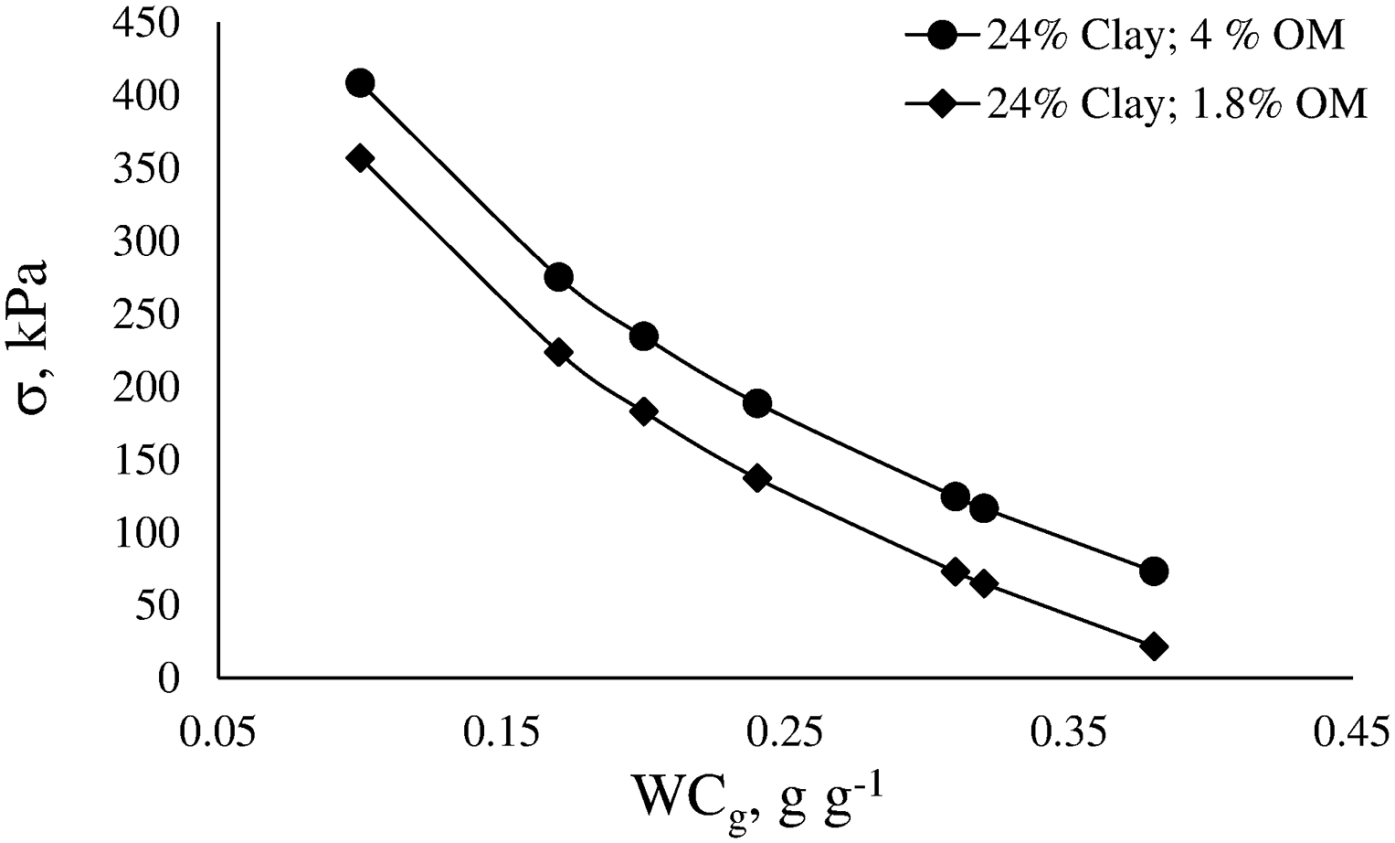
Variation of pre-consolidation pressure (σ) as function of water content (WCg) for two soil with similar clay content and different organic matter (OM) content.

**Fig 10:**
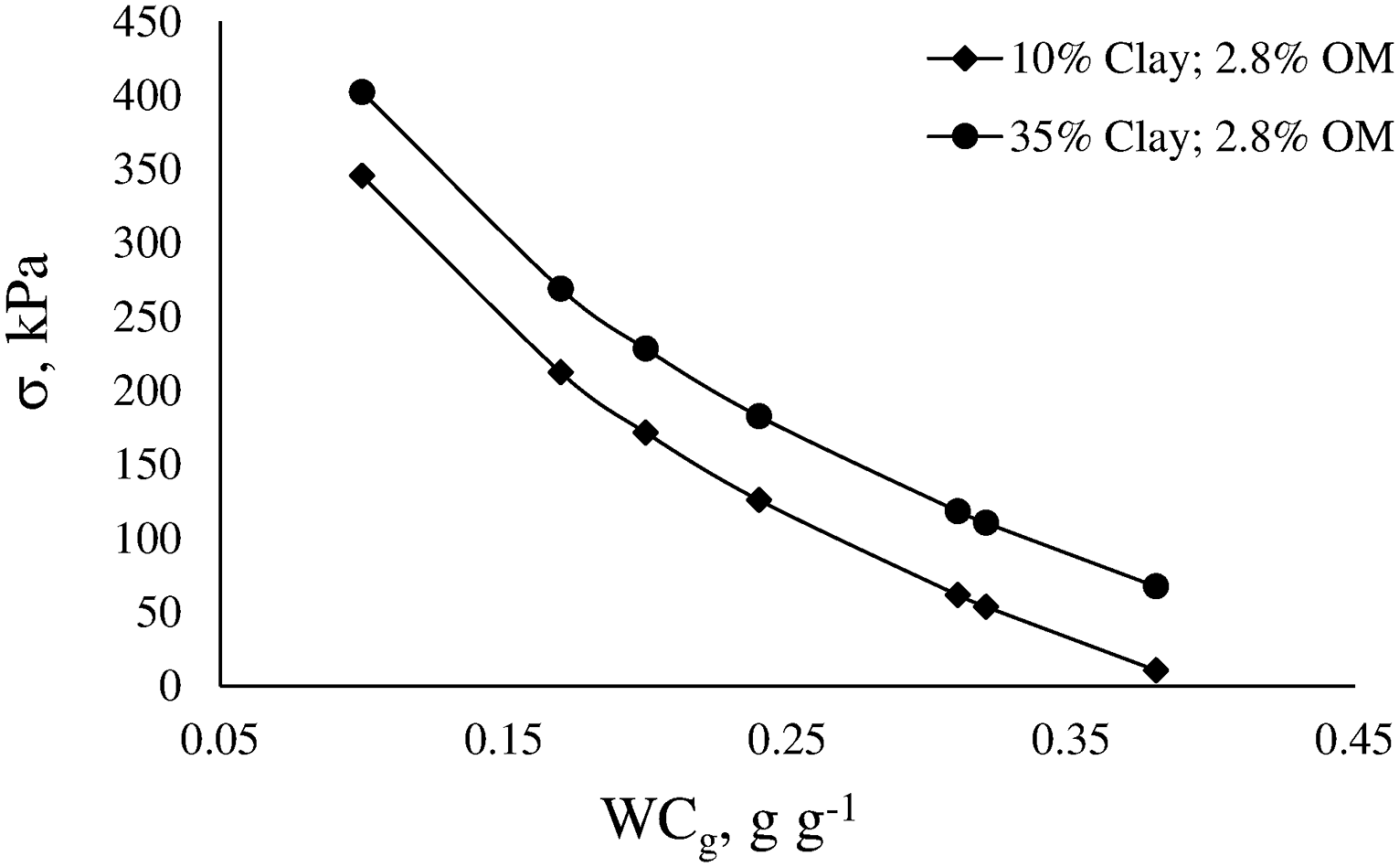
Variation of pre-consolidation pressure (σ) as function of water content (WC_g_) for two soil with similar organic matter (OM) content and different clay content.

Figs 9 and 10 show the effects of the studied variables on σ. The effects of all variables were nonlinear.

For the soil with 4% of OM and WC_g_ = 0.26±0.06 (Fig 9), σ varied between 116 and 234 kPa, while for the soil with 1.8% of OM and WC_g_ = 0.26±0.06, σ varied between 65 and 183 kPa. These results show that in deteriorated soils (low OM), if soil water content is not taken into account, traffic by common tractors and other machines would cause further compaction to the soil.

On the other hand, for the soil with 35% of clay and WC_g_ = 0.26±0.06 (Fig 10), σ varied between 110 and 228 kPa, while for the soil with 10% of clay and WC_g_ = 0.26±0.06, σ varied between 54 and 172 kPa. For the same water content, as clay content increases, the bearing capacity of the soil also increases.

These results highlight the importance of the clay fraction to increase soil strength and, as consequence, to allow traffic of common machines without causing further compaction. In addition, the content of clay seems to be more important than the type of clay as a determinant of the soil bearing capacity for a given water content. However, it is very important to note that once overcome the σ value, clayed soils will be more prone to undergo additional soil compaction due to they have higher CI [16,17]. In addition, soil bearing capacity is strongest dependent on WC_g_ at the time of applying stress. Therefore, WC_g_ should be measured and pressure applied to the soil, either from animals or agricultural machinery, should be controlled to avoid further compaction.

Compression tests may produce data that are useful not only to determine σ and CI but also to develop general models that allow estimating changes in Bd due to the applications of external stress. A review of such models have been made by Défossez and Richard [58], although other were developed after by Keller et al. [59], Keller et al. [60] and more recently by Keller et al. [9].

Eq 7 [38] was used to fit the data of this study. Initially, the model failure to converge because some parameters were not different from 0. Thus, parameters for soil water content were removed and then reintroduced individually. After that, the possible effect of texture in all parameters was checked. Results are shown in Table 7.

**Table 7:** Results of the nonlinear regression model for the estimation of soil bulk density (Bd). *LnBd* = *Ln*(*Bd*_*0*_ × *Bd*_*n*_) − [(*a* + *a*1 × *clay*) + (*b* × *σ*_*a*_) + (*c* × *Bd*_*c*_ × *Bd*_0_)] × [(1 − exp(− *d* × *σ*_*a*_ × (1 − *θ*^2^)))].

Parameter	Estimated Value±SE	Confidence limits	
		Lower limit	Upper limit
**Bd**_**0**_	1.35±0.005	1.348	1.368
**a**	-0.05**±**0.010	-0.075	-0.033
**a1**	-0.003**±**0.0003	-0.004	-0.002
**b**	-0.086**±**0.006	-0.099	-0.073
**c**	14.524**±**1.511	11.558	17.490
**d**	0.369**±**0.019	0.330	0.407

Bd: soil bulk density (g cm^-3^); Bd_0_ is Bd at zero applied stress (g cm^-3^); Bd_n_: Bd_i_/average Bd; Bd_c_: ((Bd_n_-1)_*_Bd_0_); Bd_i_: initial soil bulk density (g cm^-3^); σ_a_: applied stress (kPa); θ: (1-θ_SD_); θ_SD_: water saturation degree; *a*, *a1*, *b*, *c*, and *d*, are the estimated parameters. *F*-value of the model = 14224; significant at *P* < 0.0001 probability level; *R*^*2*^ = 0.88.

All parameters were significant and the model accurately predicts Bd of the soils studied (R^2^ = 0.88). Although parameters *a*, *a1*, *b*, *c*, and *d* were correlated with one another, the values of the correlation coefficient (r) were lower than ± 0.99, which indicates the model contains too many parameters [38].

The value of the parameter *g* that resulted in better model fit was 2, which agrees with the value suggested by McNabb and Boersma [38]. The value of *g* = 2 shows that the effect of degree of saturation on the final value of Bd was nonlinear.

The inclusion of the effects of Bd_i_, clay content and water content (expressed as saturation degree) allow improving the model fit and decreasing the MSE. Parameter *a* was significantly correlated with *b* (r = 0.50), showing the effect of clay content on soil response to the σ_a_, while *c* was correlated with *d* (r = 0.66), showing the effect of Bd_i_ and soil water content at the time of applying the stress (σ_a_).

**Fig 11:**
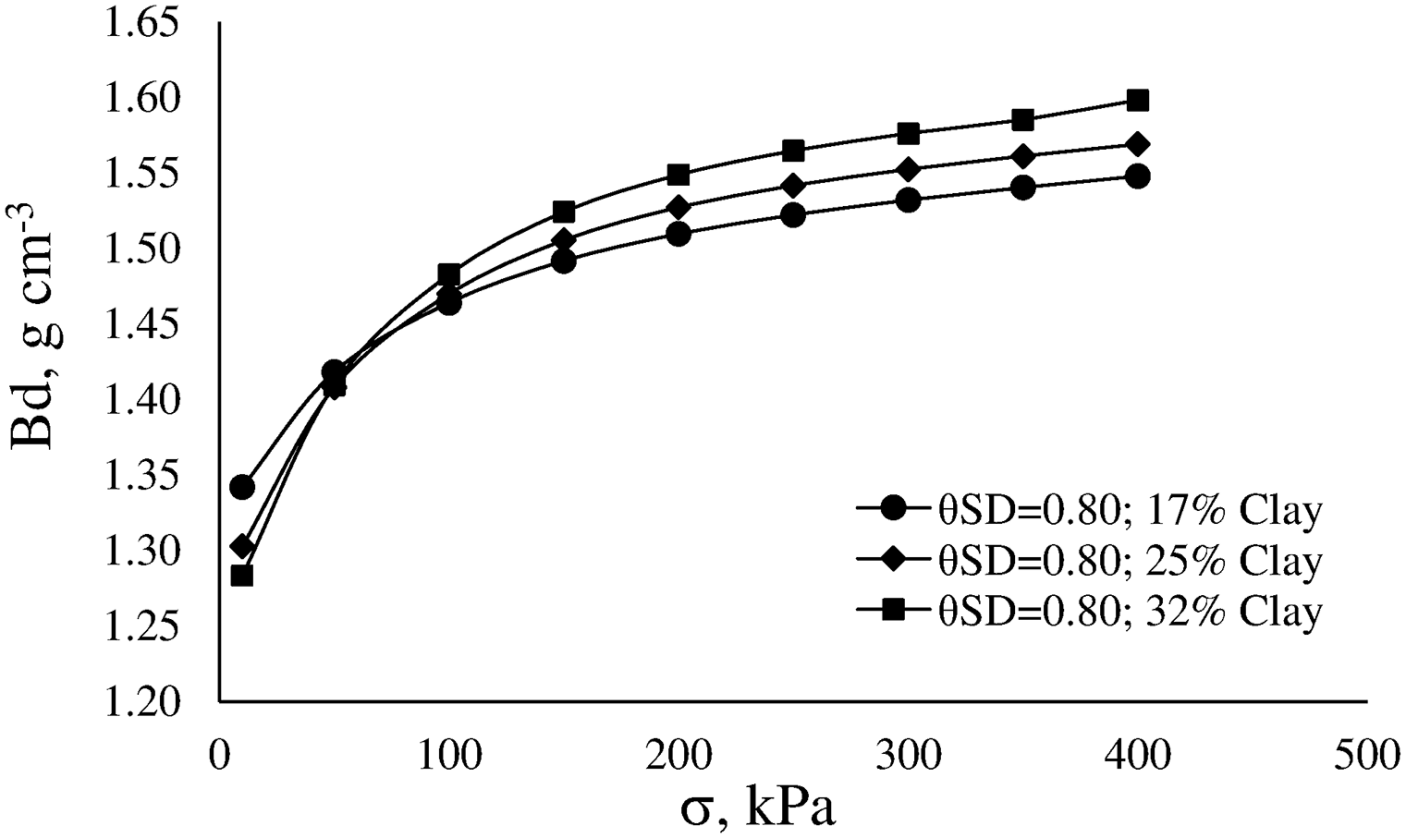
Final Bd as function of the applied stress (σ_a_) for equal levels of water saturation degree (θ_SD_) and different clay content.

The obtained model (Table 7) was used to shows the influence of clay and water content on soil compressibility (Fig 11).

The compression curves of three soils with similar water saturation degree (Fig 11) show that soil compressibility changes with increasing σ_a_. These changes are conditioned by the initial state of soil compaction and soil texture. Similar behavior may be verified when changing soil water content. The higher the initial Bd, the lower the soil deformation is for the same applied load until reaching a value of about 50 kPa. Once exceeded this value, soil compressibility increases with increasing clay.

Soil compressibility may be described by three stage following the increase of σ_a_ [60]. In the first stage, soil volume changes correspond to elastic deformation and the σ_a_ is lower than σ. In the second stage, soil volume changes correspond to the reduction of volume of air-filled pores. In the third stage, soil volume changes correspond to the reduction of volume of water-filled mesopores. This last stage is associated with the plastic deformation of the soil. Moreover, the changes of volume at the three stages are controlled by the gradation, shape and orientation of soil particles as well as the interactions forces between soil particles, binding agents and soil water.

For the studied soils, volume changes at the first stage were mainly conditioned by differences in soil structure (differences in Bd_i_) while changes at the second and third stages were conditioned by Bd_i_ and other intrinsic soil properties. Tang et al. [61] found that as clay content increased, air-water interaction forces also increased. As a result, differences in the slope between the second and third stage of the compression curve were more evident in soils with high clay content. The results of the present study confirm the findings of Tang et al. [61].

**Fig 12:**
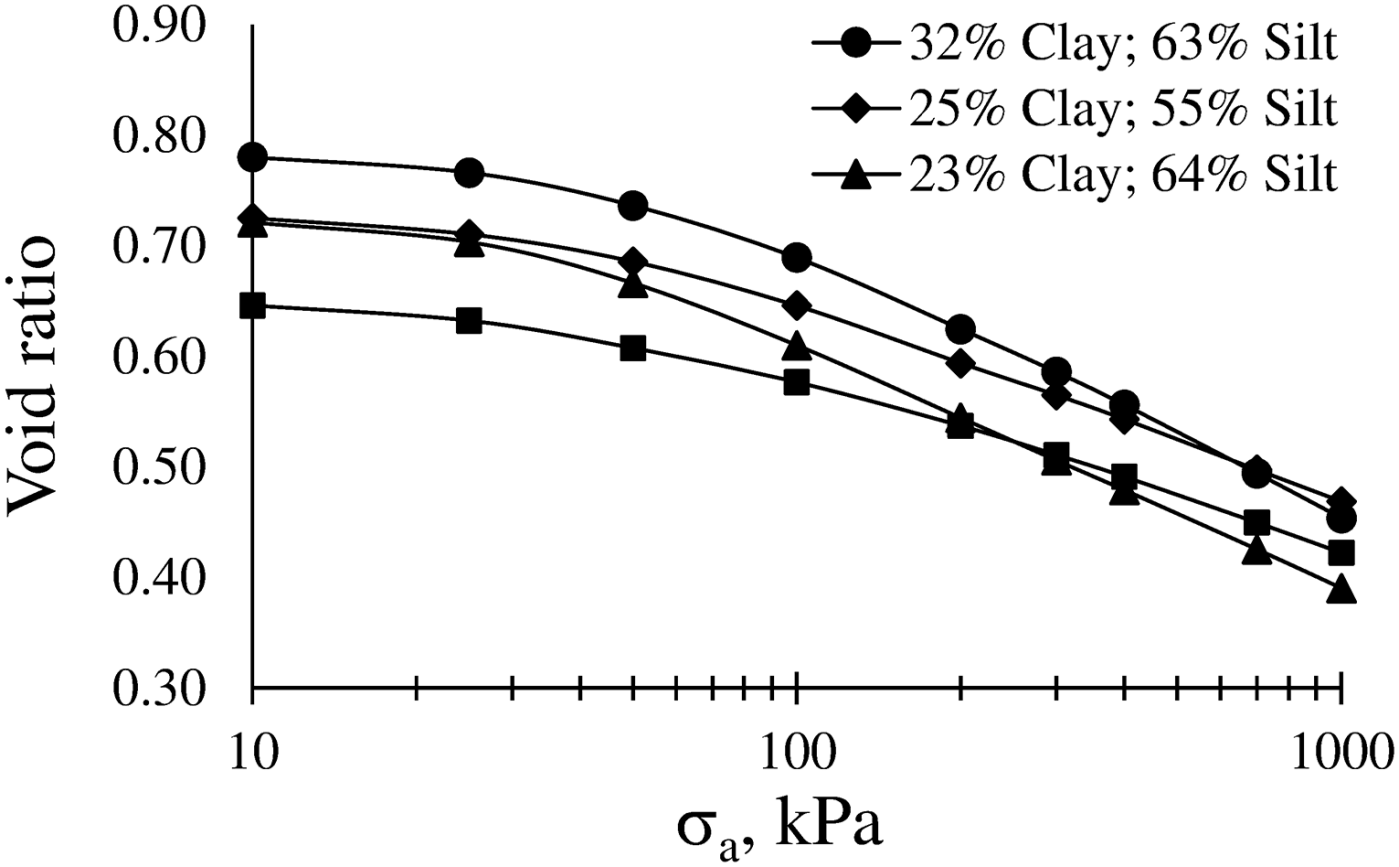
Relationships between void ratio and applied stress (σ_a_) of four Argiudolls: Humboldt series (clay = 32%, silt = 63%), Recreo series (clay = 25%, silt = 55%), Rafaela series (clay = 23%, silt = 64%), Santa Isabel series (clay = 17%, silt = 48%).

Fig 11 shows that in the most common operating pressure range of the agricultural machinery (80–250 kPa), differences in the slope (ΔBd/Δσ_a_) of the compression curves increase between soils with increasing clay content. Silt content did not significantly affect the compression curve model. Despite this result, the influence of silt on the compressive behavior of the studied soils should not be absolutely disregarded as suggested by Fig 12 and especially if one considers that silt strongly affected CI. Fig 12 shows the measured compression curves of four soils at field capacity water content (ψ = -10 kPa).

These curves seem to confirm that silt content in some way affects soil behavior. The soil with the lower contents of clay and silt shows the lower deformation whereas the opposite is observed for the soil with the contents of clay and silt. The two soils with similar content of clay show similar behavior until σ_a_ was about 50 kPa. After that, the soil with higher content of silt shows a curve similar to that of the soil with highest content of clay. The application of pressures values that exceed σ may cause the collapse of the fine fraction of silt. Especially under wet conditions, not only clay but also the loose particles of silt can be displaced and reorganized into closer positions. This process would explain the great change of volume experienced in the second and third stages of the studied soils that have high values of silt content. These results also support the findings of Días-Zorita and Grosso [24], whom have pointed out that soil compactability of the Pampas soils is conditioned by the content of silt. These findings suggest this point deserves further analysis to better understand the mechanical behavior of the Flat Pampa soils. In addition, the results highlight the importance of measuring soil water content and controlling the pressure applied to the soil by the agricultural machinery especially in the second state of the curve, which generally matches the most common work pressures.

From a practical point of view, this study allowed the development of useful PTFs to link the soil mechanical behavior and the soil quality to plants growth. For each soil condition, the compression curve may be determined as well as the soil Bd_c_ (Fig 7). The estimated Bd_c_ value can be introduced in the model of the compression curve (Table 7) to determine the maximum acceptable pressure to be applied during tillage operations. Soil conditions shown in Fig 11 are used as an example. The soil with 32% of clay content has Bd_c_ = 1.48 g cm^-3^ whereas the soil with 25% of clay has Bd_c_ = 1.55 g cm^-3^. Thus, pressure applied to the soil by the agricultural machinery must be less than 150 kPa for the former and less than 300 kPa for the latter in order to maintain adequate soil conditions to plant growth. In addition, σ_a_ can be set taking into account safer values of Bd, which in turn could be set by considering a percentage of the Bd_c_ (e.g. 85%) as maximum accepted Bd. In this way, the amplitude of the LLWR could be maintained in proper values for each specific condition of climate-crop-soil.

Furthermore, the PTFs may be used as input parameters of dynamics simulations models, such as of the Keller et al. [9], to estimate the LLWR changes of superficial layers or the subsoil that could happen due to the tillage operations and the traffic of agricultural machinery.

## Conclusion

This study shows that the quality and the mechanical behavior of the most productive soils of the Pampas Region of Argentina can be estimated based on pedotransfer functions. The assessed functions of soil water retention and soil resistance to penetration allow the estimation of the least limiting water range and the soil critical bulk density for plant growth. The former is mainly related to soil bulk density, clay and organic matter content whereas the latter is related to clay content. The assessed functions of the compression index, pre-consolidation pressure and compression curve shows that soil texture, bulk density, water and organic matter contents are the properties that control the mechanical behavior of the soils studied. The maximum acceptable pressure to be applied during tillage operations can be calculated by introducing the estimated value of soil critical bulk density for plant growth in the model of the compression curve. Thus, the developed pedotransfer functions provide a useful tool to link the soil quality to plants growth and the soil mechanical behavior.

## Supporting Information

S1 Dataset File with all data. (RAR)

S1 Fig Values of error (measured-estimated) as function of clay content for water content at field capacity (FC) and wilting point (WP). Values of six additional soils are included.
(TIFF)

S2 Fig Estimated values versus measured values of soil resistance (SR) of the soils studied for water content at field capacity and wilting point. (TIFF)
