# Supplementary figures and images for: Physical Quality Indicators and Mechanical Behavior of Agricultural Soils of Argentina
Source: PLoS One. 2016 Apr 21;11(4):e0153827. doi: 10.1371/journal.pone.0153827 (PMC4839615; doi:10.1371/journal.pone.0153827)

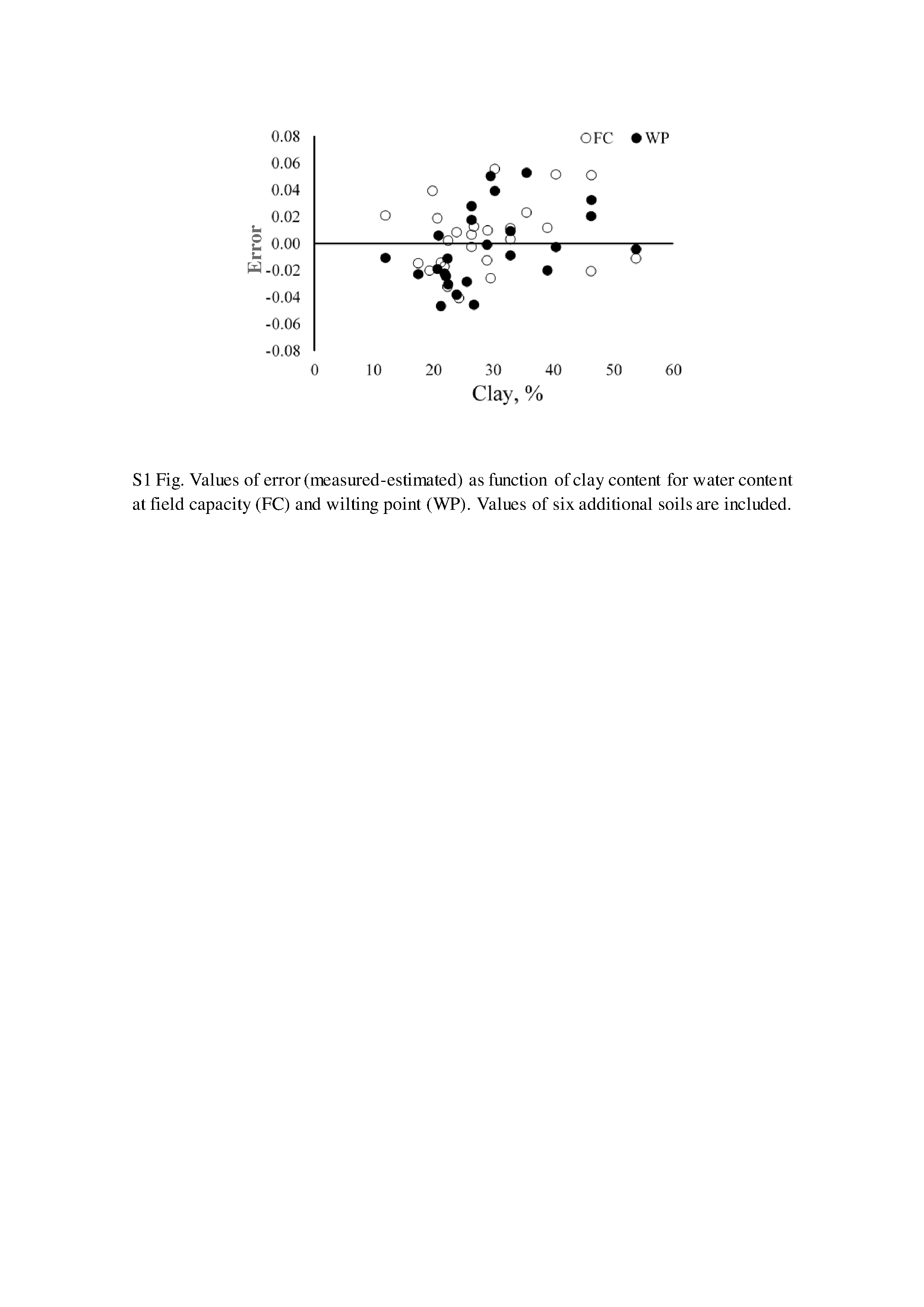

Supplement: S1 Fig — Values of six additional soils are included. (TIFF) [file pone.0153827.s002.tiff]

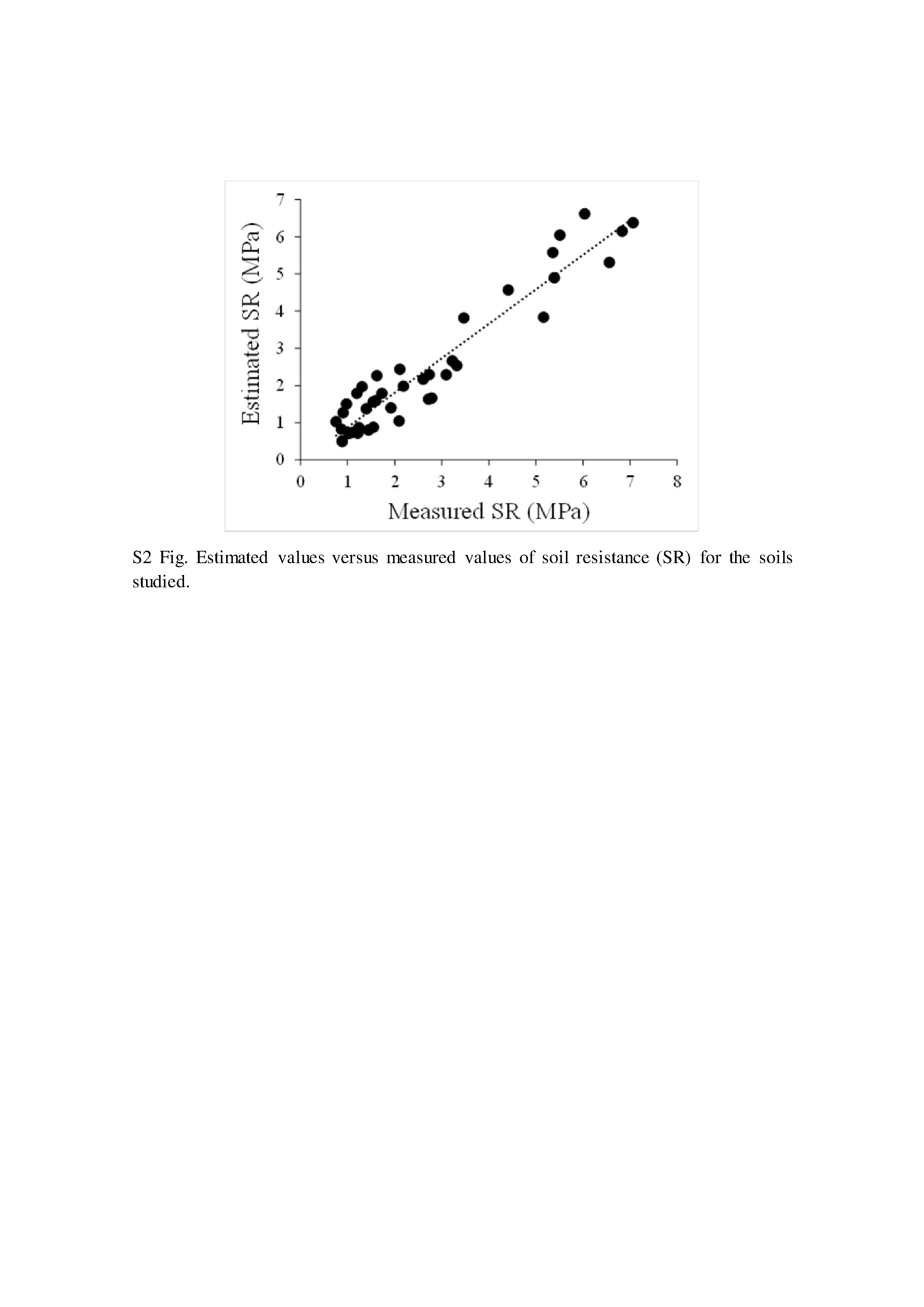

Supplement: S2 Fig — (TIFF) [file pone.0153827.s003.tiff]
